# Feasibility and Safety of Argon Cold Plasma Use as an Adjunctive Treatment for Corneal Disease in Dogs, Cats and Small Mammals: A Prospective Clinical Study

**DOI:** 10.1111/vop.70145

**Published:** 2026-01-30

**Authors:** Petr Soukup, Ingrid Allgoewer

**Affiliations:** ^1^ Animal Eye Practice Berlin Germany

**Keywords:** corneal infection, corneal perforation, keratomalacia, plasma pen, spontaneous chronic corneal epithelial defect, stromal ulcer

## Abstract

**Objective:**

Evaluation of the feasibility of argon cold plasma (ACP) use in corneal diseases in clinical practice and assessment of short‐ and long‐term safety in dogs, cats, and small mammals.

**Animals Studied:**

Client‐owned animals.

**Procedure:**

ACP was performed in animals with corneal diseases using the kINPenVET (Neoplas GmbH, Germany) under topical anesthesia as an adjunct to standard treatment. Early (< 30 days) and long‐term (> 30 days) adverse reactions and events were monitored.

**Results:**

303 eyes in 281 animals were treated with ACP (263 dogs, 8 cats, 10 small mammals) with the following conditions: spontaneous chronic corneal epithelial defects (SCCED, *n* = 178), infected/infiltrated ulcers (*n* = 76), keratomalacia (*n* = 22), corneal perforation (*n* = 20), other (*n* = 7). Treatment under topical anesthesia was well tolerated with only a few animals showing stress during the procedure (3.2%, 8 dogs, 1 cat). The average number of treatments per eye was 3.45 ± 1.74 with a median of 3 (1–9). The two direct adverse reactions observed were punctate opacities (1.3%, *n* = 4) and blepharospasm (0.7%, *n* = 2). Short‐term adverse events were: epithelial tears differing from the initial ulceration (4.0%, *n* = 12), development of stromal infiltrates (2.3%, *n* = 7), occurring/persistent keratomalacia (2.0%, *n* = 6). Long‐term follow‐up period averaged 173.1 days with a median of 117 (30–619) days. The most common long‐term adverse events were significant corneal fibrosis and pigmentation (4.0%, *n* = 12, mostly cases of dry eyes).

**Conclusions:**

ACP with kINPenVET is a clinically feasible outpatient treatment for various corneal diseases with a low number of short‐ and long‐term adverse reactions and events. It can be easily integrated into clinical treatment protocols.

## 1 Introduction

Cold atmospheric plasma has been recognized for many years as an effective regenerative therapy for the treatment of chronic wounds [1, 2, 3] and has found its way to veterinary dermatology and other areas of veterinary medicine [4, 5]. It has attracted attention due to its antimicrobial properties [6, 7, 8, 9, 10], particularly in light of increasing antibiotic and antifungal resistance [9, 10, 11, 12, 13]. Its potential for use in oncology is currently being investigated [14].

Cold atmospheric plasma is a low‐temperature ionized gas consisting of ions, electrons, reactive oxygen species, reactive nitrogen species, ultraviolet radiation, and an electromagnetic field [1, 15]. Plasma discharge reduces the viability of bacteria through ultraviolet radiation, reactive oxygen and nitrogen species, and the generation of electric current [1]. Various devices have been developed for cold plasma therapy, including plasma jets, microplasma jets, and dielectric barrier discharge devices using different gas sources, including argon, helium, and nitrogen [2].

In the field of medical and veterinary ophthalmology, the antimicrobial efficacy of cold plasma treatment has been confirmed in vitro against common ocular pathogens *P. aeruginosa, E* *. coli* , *S* *. aureus* , *S. epidermidis, C* *. albicans* , *and A* *. fumigatus* [9, 16, 17], canine isolates *P. aeruginosa, S. pseudintermedius*, *and Streptococcus canis* [7], equine isolates *A. flavus* and *Fusarium keratoplasticum* [12], and in vivo (rabbits) against *P. aeruginosa* [6], methicillin‐resistant *S. aureus* [18], and *C. albicans* [19]. The effects of cold plasma on corneal wound healing after keratectomy were studied in an ex vivo healing model for corneal tissue from dogs using air‐liquid interface culture [20] and in an in vivo healing model of corneal wound from rabbits [21].

Although its efficacy against various corneal pathogens has been extensively studied, there are only a few scientific publications on corneal safety. In vitro and ex vivo effects of cold plasma treatment on keratocytes and conjunctival fibroblasts [16], primary human corneal limbal epithelial cells and donor corneas [9], as well as corneal tissue from dogs [20], did not reveal any safety concerns. Some in vivo corneal models described ACP as a safe treatment method for the cornea from New Zealand White Rabbits [18, 21]; however, these studies did not focus on toxicity and safety.

Recently, a first in vivo study on ocular toxicity and safety in New Zealand White Rabbits using multimodal imaging was conducted, showing mild and transient changes in corneal opacity [22]. The only data on ocular safety in patients come from four volunteers with refractive corneal infections who showed no adverse reactions after cold plasma therapy [9]. Based on the ex vivo results, Dick et al. discussed the transferability of their ex vivo findings on the cornea to in vivo and advocated a prospective clinical study in dogs [20].

The purpose of this prospective study is to assess the feasibility of the ACP use on diseased cornea in dogs, cats, and small mammals in a clinical setting and to evaluate short‐ and long‐term clinical safety outcomes. Concerning safety, both adverse reactions of ACP treatment and adverse events following ACP treatment are documented. An adverse reaction is defined as a harmful and unintended response to treatment. An adverse event is defined as any undesirable medical event that is not necessarily causally related to treatment [23].

## 2 Materials and Methods

### 2.1 Animals and Ophthalmic Examination

All animals presented to the Animal Eye Practice Berlin, Germany with one of the following diagnoses were eligible for inclusion in the present study: (1) Animals with spontaneous chronic corneal epithelial defects (SCCED). (2) Animals with an infected or infiltrated stromal ulcer. (3) Animals with keratomalacia. (4) Animals with corneal perforation due to infectious keratopathy or keratomalacia that could not be treated surgically. (5) Animals with epithelialized deep stromal ulcers or descemetocele without sufficient neovascularization. (6) Rabbits with an indolent ulcer.

Owners of the above‐mentioned animals were informed about the ACP, and their consent was obtained prior to the first ACP treatment. Also, animal owners or owners' representatives provided written consent for the publication of data and images. This prospective study was conducted in accordance with GERVO guidelines and approved by the competent state authority (Landesamt für Gesundheit und Soziales, Berlin, Germany) under registration number StN° 032–2023.

All animals underwent an ophthalmic examination including a slit lamp biomicroscopy (Kowa SL‐17, Kowa Ltd., Japan), indirect ophthalmoscopy examination (Video Omega 2C, Heine Optotechnik GmbH & Co. KG, Germany) and fluorescein staining (Fluoro‐Touch, Madhu Instruments Pvt. Ltd., India) as well as thorough photographic documentation. In most cases, the Schirmer tear test I (Tear Touch Blu, Madhu Instruments Pvt. Ltd.) and rebound tonometry (Tonovet or Tonovet+, iCare Oy, Finland) were performed as well. A bacterial culture was performed in all cases of keratomalacia and stromal ulceration, as well as in SCCED cases in brachycephalic dogs.

### 2.2 Argon Cold Plasma Treatment

Before ACP, topical anesthesia (proxymetacaine hydrochloride; Proparakain‐POS 0.5%, Ursapharm, Germany) was administered every 3–5 min over a total period of at least 20 min. The animals were manually restrained on the examination table by a veterinary technician. Before the procedure, the eye was rinsed with sterile saline. ACP was performed using kINPen VET (Neoplas VET, Neoplas GmbH, Germany) as the plasma source, as previously described [7]. The device was set on a gas flow rate between 4.6–4.8 L/min, similar to 5 L/min reported by Reitberger et al. [9]. The corneas of the animals were irradiated for 30–90 s in a meandering motion over the lesion; according to the manufacturer's recommendations, the total dosage should be approximately 60 s per 1 cm^2^.

**FIGURE 1:**
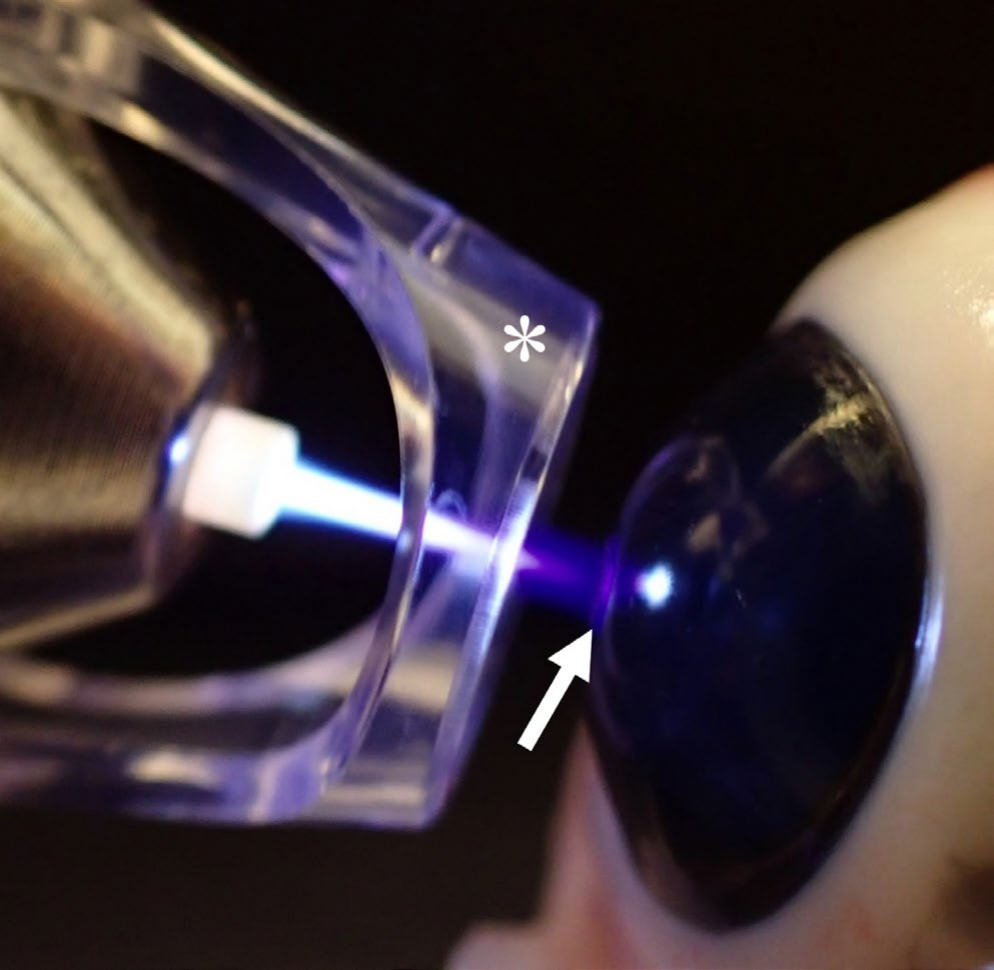
Argon cold plasma treatment procedure: The plasma pen was held in an angle of 90° to the cornea surface; plastic plasma pen spacer (marked with asterisk) was positioned approximately 2–3 mm away from the corneal surface, and the plasma jet interface tip lightly touched the treated cornea, enabling continuous plasma treatment in conductive mode (visible reaction with cornea tissue, where small plasma currents leap over to the adjacent cornea, marked with an arrow). Demonstrated on a pig's cadaver eye.

The plasma pen was held at an angle of 90° to the cornea surface; the distance between the plasma pen spacer and the cornea was approximately 2–3 mm, and the tip of the plasma jet interface tip was slightly touching the treated cornea (Figure 1) allowing for continuous plasma treatment in conducting mode (visible reaction with cornea tissue where little streams of plasma leap over to neighboring cornea) as reported by Dick et al. [20]. During the procedure, the cornea was constantly irrigated with sterile saline, slowly dropped through a 22‐G catheter. If the cornea appeared dry, the eyelids were blinked once or twice during the procedure.

To be fully effective, according to the manufacturer's advice, the ACP treatment was repeated every 2–6 days until the cornea had healed. After the procedure, hyaluronic acid eye drops (Hylo‐Vision Gel Multi; OmniVision GmbH, Germany) as well as atropine eye drops (Atropin‐POS 0.5%, Ursapharm) were applied unless contraindicated.

In this pilot study in small animals to evaluate the feasibility and safety in clinical practice, treatment with ACP was never used as a monotherapy, but always as an adjunct to standard therapy for the respective disease. In particular, diamond burr debridement was used as a standard therapy for SCCED in this study.

### 2.3 Feasibility, Short‐Term Safety and Monitoring of Adverse Events

The animals were observed for procedural distress or immediate signs of discomfort during and directly after the ACP treatment. The owners observed and reported on any adverse events between treatments. In the short‐term safety phase, all adverse reactions to ACP treatment and adverse events occurring from the first ACP treatment until 30 days after the first ACP treatment were recorded.

### 2.4 Long‐Term Safety and Monitoring of Adverse Events

All the animals observed for more than 30 days after the first ACP treatment were evaluated for any long‐term adverse reaction to ACP treatment and all adverse events following ACP treatment.

All adverse events were carefully investigated for a possible association with ACP and the results were discussed. Neither worsening of the keratoconjunctivitis sicca nor progression of cataracts diagnosed before the first ACP treatment was included in the list of adverse events.

### 2.5 Statistical Analysis

Descriptive statistical analysis of the dataset, feasibility, short‐term and long‐term safety was performed using Microsoft Office Excel (Microsoft Corporation, United States).

## 3 Results

**FIGURE 2:**
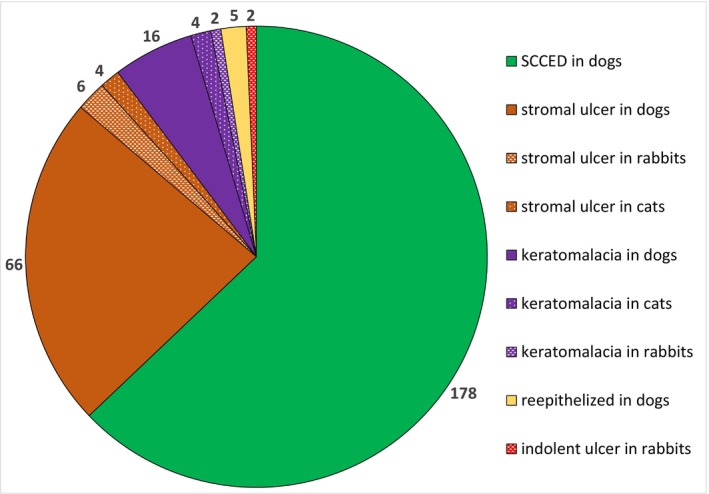
Diagram of conditions and animal species included in the study.

During the twenty‐month study period (July 2023 to February 2025), a total of 303 eyes from 281 animals were treated with ACP, including 285 canine eyes (263 dogs), eight feline eyes, nine rabbit eyes and one guinea pig eye. There were 54.5% left eyes (*n* = 165) and 45.5% right eyes (*n* = 138) represented. Most of the animals were diagnosed with SCCED (*n* = 178), followed by infected or infiltrated stromal ulcer (*n* = 76), keratomalacia (*n* = 22), corneal perforation (*n* = 20), epithelized deep stromal ulcer or descemetocele without sufficient neovascularization (*n* = 5) and rabbit indolent ulcer (*n* = 2). The diagram of the presenting conditions and species is shown in Figure 2. In dogs, the most common breeds were French Bulldog (*n* = 130), Pug (*n* = 18), Boxer (*n* = 14), Shih‐Tzu (*n* = 11), English Bulldog (*n* = 9), Bolognese (*n* = 8), Yorkshire Terrier (*n* = 6), Chihuahua (*n* = 5), Old English Bulldog (*n* = 4) and Staffordshire Bullterrier (*n* = 4) as well as mixed‐breed dogs (*n* = 13). The following breeds of cats were represented: domestic shorthair (*n* = 2), British shorthair (*n* = 2), Persian (*n* = 2), Bengal (*n* = 1) and Norwegian Forest cat (*n* = 1). Various breeds of dwarf rabbits were represented among the rabbits.

The average number of ACP treatments was 3.45 per eye, with a median number of 3 (1–9) treatments. A total of 1044 ACP treatments were performed. Short‐term follow‐up was available for all of the eyes included in the study (303 eyes from 281 animals); animals with no follow‐up were excluded from the study. Long‐term follow‐up was available for 221 eyes with an average duration of 173.1 days and a median duration of 117 (30–619) days.

In terms of feasibility, ACP treatment was a straightforward outpatient procedure, with most animals having no difficulty remaining still throughout the entire procedure. 3.2% of the animals (*n* = 9, 3.0% of dogs *n* = 8; 12.5% of cats *n* = 1) experienced some distress during the procedure. Two dogs (French Bulldogs) and one cat (British Shorthair) were unsettled by the airflow on the surface of their eyes. Three dogs (Australian shepherd, German shepherd and mixed‐breed dog) were very restless on the treatment table during the first procedure, but all subsequent treatments were without any complications. Two dogs (French Bulldog, Bolognese) were extremely restless throughout all of the treatments, one of them (the French Bulldog) even experiencing breathing difficulties during the second ACP treatment. One very aggressive Boxer became so violent during the procedure that the ACP treatment had to be discontinued. Overall, the procedure was very well received by the treating veterinarians and could be easily incorporated into the clinical outpatient treatment protocols.

**FIGURE 3:**
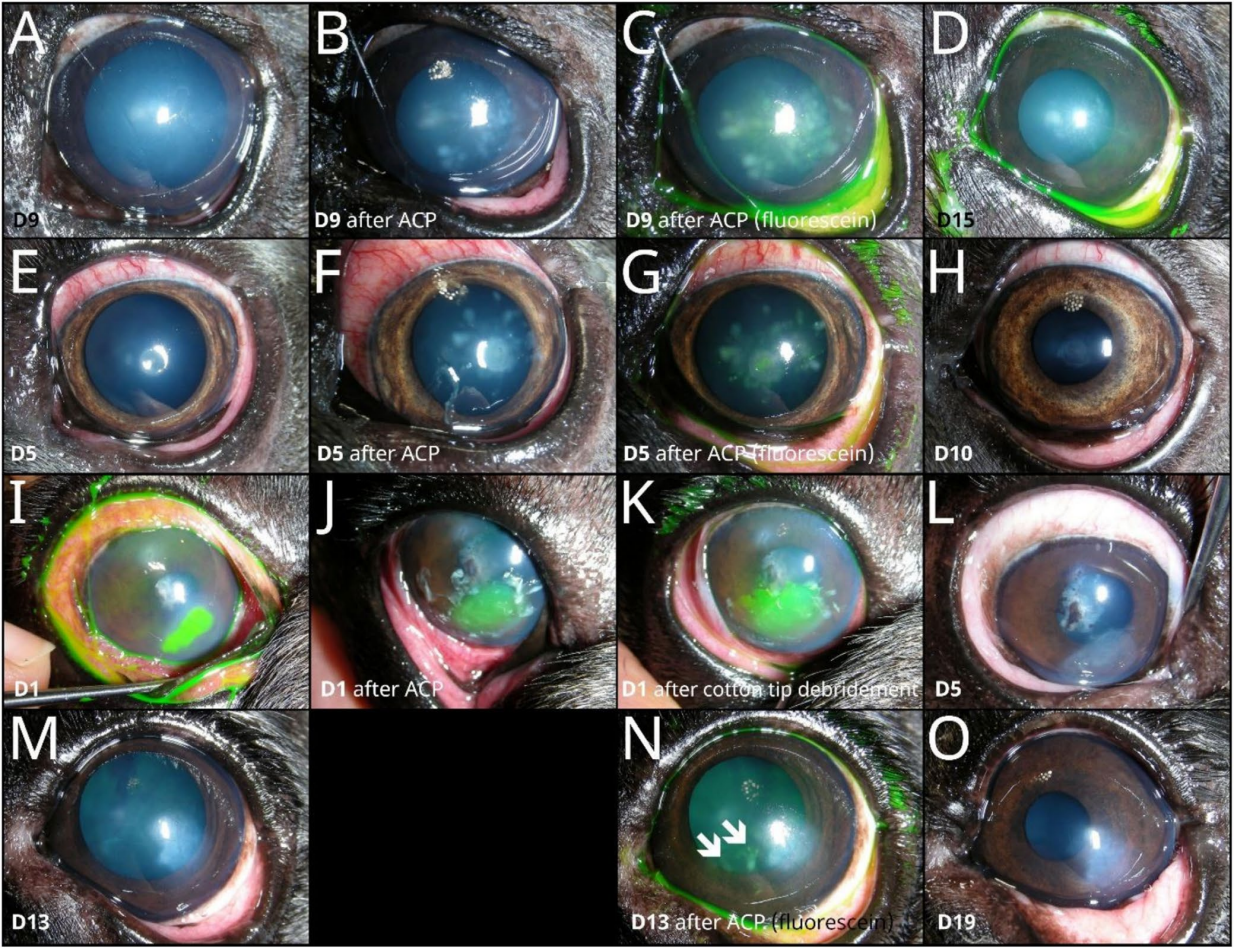
Adverse reaction seen immediately after ACP treatment observed in four canine eyes (4/285, 1.4%): An eight‐year‐old French Bulldog with SCCED showed fluorescein‐negative epithelial and anterior stromal whitish spots after the third ACP treatment (A–D). A four‐year‐old French Bulldog with a small mid‐stromal infected ulcer showed whitish spots in the anterior stroma after the second and third ACP treatments (E–H), which were centrally fluorescein positive (G). A six‐year‐old French Bulldog with a large infected ulcer in the anterior stroma developed whitish epithelial scabs (J) at the edges of the ulcer after the first ACP treatment, which could be removed immediately afterwards with a cotton swab (I–L). A five‐year‐old French Bulldog presenting with SCCED showed multiple fluorescein‐negative epithelial and anterior stromal whitish spots (arrows in N) after the third ACP treatment (M–O). The first column shows the cornea immediately before ACP treatment (A, E, I, M), the second column after ACP treatment (B, F, J), the third column after ACP treatment with fluorescein staining (C, G, K, N) and the fourth column shows the cornea at the next visit (D, H, L, O).

Short‐term adverse reactions after the ACP procedure occurred in two forms. Firstly, immediately after the ACP treatment, whitish spots were observed in the corneal epithelium and anterior stroma in 1.4% of dogs (*n* = 4), all of which were French Bulldogs (Figure 3). In two eyes, these did not retain the fluorescein stain (Figure 3C,N); in one case with anterior stromal ulceration, they appeared as scabs on the adjacent epithelium and could be removed with a cotton swab (Figure 3J), and in one dog, the spots were centrally fluorescein‐positive (Figure 3G). All of these spots had disappeared before the next ACP treatment (Figure 3D,H,L,O). Secondly, 0.7% of the dogs (*n* = 2) suffered from blepharospasm and pain several hours after the procedure, according to the owner. In one dog (mixed‐breed), this was caused by inadequate topical anesthesia before the ACP procedure and occurred only once; the other dog (French Bulldog), an extremely sensitive dog with atopy, reportedly experienced pain after all three sessions. All details of adverse reactions are listed in Table S1.

**FIGURE 4:**
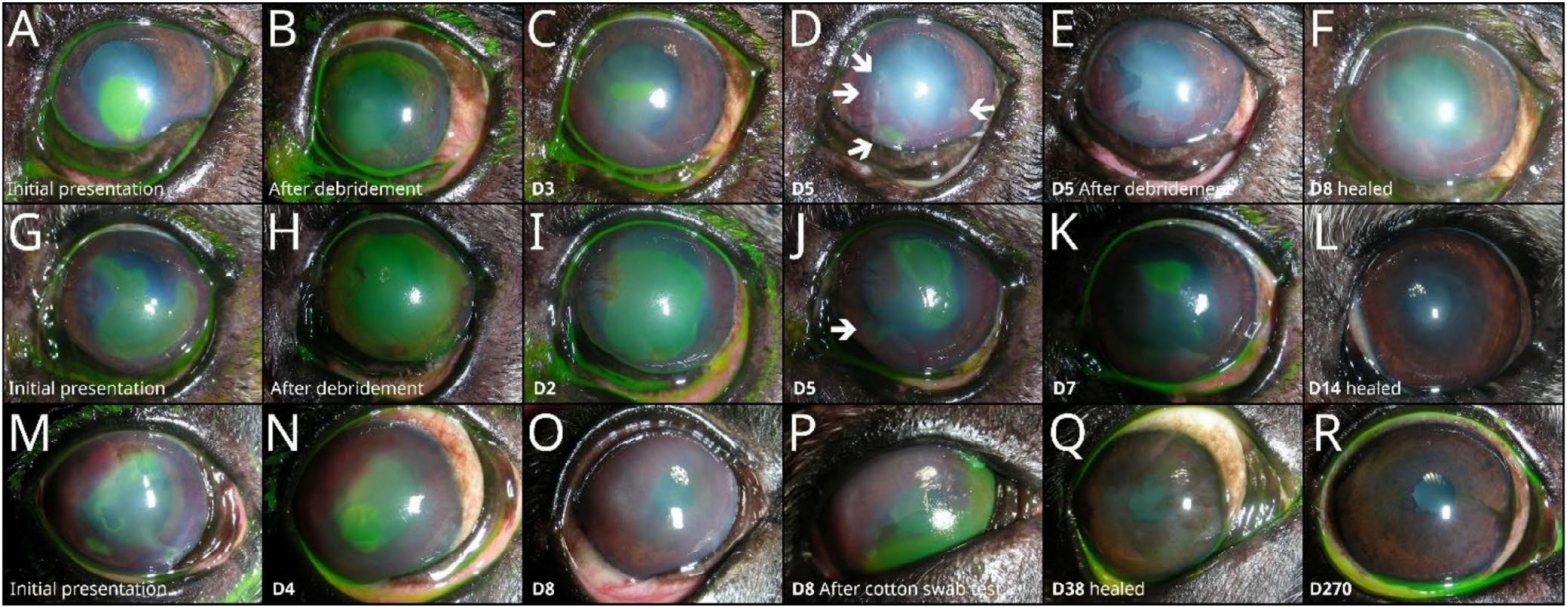
Short‐term adverse events in dogs with SCCED: 6.7% of SCCED dogs (*n* = 12) developed either epithelial tears after complete re‐epithelization (P) or peripheral epithelial tears in centrally unhealed SCCED (D, J). A nine‐year‐old French Bulldog (A–F) with SCCED showed several new fluorescein‐positive areas (arrows in D) 5 days from the beginning of the therapy and after two ACP treatments. A nine‐year‐old French Bulldog (G–L) with SCCED showed a new fluorescein‐positive area 5 days after the beginning of the therapy and after two ACP treatments (arrow in J). An eight‐year‐old French Bulldog (M–R) with SCCED showed a massive epithelial tear in the fluorescein‐negative cornea (cotton swab test) 8 days from the start of therapy and after two ACP treatments (P).

The following short‐term adverse events were observed. First, 2.5% of dogs (*n* = 7) treated for SCCED developed stromal infiltration during therapy after one or more ACP treatments (details in Table S2), but these healed without complications. Second, one French Bulldog (0.4%) treated for SCCED developed keratomalacia after the third ACP treatment, which could not be stopped by any conservative means, including corneal cross‐linking therapy. The eye was later perforated and was enucleated at the owner's request. Thirdly, in 4% of animals with keratomalacia or infected stromal ulceration (*n* = 2 dogs and 2 rabbits), the progressive process could not be stopped by ACP treatment, and animals later developed a descemetocele and/or corneal perforation. Finally, the most common adverse event in 6.7% of dogs with SCCEDs (*n* = 12) was either epithelial tears after complete re‐epithelization or peripheral epithelial tears in centrally unhealed healed SCCED (Figure 4). All details of short‐term adverse events are listed in Table S2.

In 20 eyes (out of 221 in long‐term follow‐up) of 20 animals, the following long‐term adverse events were observed after ACP treatments: (1) The most common adverse event was persistent corneal fibrosis and pigmentation despite intensive therapy after re‐epithelization in 5.4% of dogs (*n* = 12, including four French Bulldogs, three Pugs and three Shih‐Tzus). (2) Three dogs (1.4%) developed glaucoma in the eye treated with ACP (163 days, 310 days and 451 days after ACP), one of which also developed retinal detachment as well. (3) One dog developed exophthalmos and retrobulbar disease 400 days after ACP treatment. (4) One dog (Boxer) developed canine endothelial dystrophy 410 days after ACP treatment. (5) One dog developed episcleritis 108 days after ACP treatment. (6) One dog developed SCCED 298 days after ACP treatment. (7) A dog that was treated for chronic myositis of the left masticatory muscle was diagnosed with trigeminal nerve paralysis after ACP treatment and healing of the underlying disease, a melting ulcer. Full details of long‐term adverse events can be found in Table S3.

## 4 Discussion

In this report, we present a first large‐scale observational clinical study on the safety of ACP use in the diseased cornea in companion animals. To date, there have been only a few in vitro and ex vivo cell and tissue studies describing ACP as a safe treatment for various human ocular surface cells and human corneal tissue [9, 16]. A case series of four volunteer patients described no adverse reactions associated with ACP treatment [9]. Several in vivo corneal models described ACP as a safe treatment method for the corneas of New Zealand White Rabbits: A model of corneal wound healing [21], a model of methicillin‐resistant *Staphylococcus aureus* keratitis [18], and an assessment of ocular toxicity after ACP treatment using multimodal imaging [22].

Dick et al. recently published the first report of the effect of ACP treatment with the kINPen VET plasma pen on an ex vivo healing model of corneal tissue of dogs using air‐liquid‐interface culture [20]. Their results showed that 2 or 5 min of ACP treatment had no detectable negative effects on ex vivo healing of epithelial wounds or on corneal tissue itself. In addition, the corneal stroma showed a higher number of Ki‐67 positive stromal cells after a two‐minute treatment, indicating increased cell proliferation compared to five‐minute therapy. The authors therefore conclude that two‐minute ACP treatment can promote cell proliferation.

Our data showed that ACP application is very easy to perform and that the tested protocols can be integrated into everyday outpatient care. Most animals tolerated the ACP application well. Of the 3.2% of animals that experienced some distress during treatment (*n* = 9), six became accustomed to ACP treatment after one or two sessions. Only 1% of all treated animals (*n* = 3, Bolognese, French Bulldog, and Boxer) either exhibited aggressive behavior during therapy, so that the next session was not carried out (Boxer), or did not get used to ACP treatment even after several sessions.

This study identified two types of adverse reactions that are directly related to the ACP treatment.

First, four dogs developed whitish spots in the corneal epithelium and anterior stroma, some of which were fluorescein‐positive and some of which appeared as whitish epithelial crusts at the edges of the stromal ulcers. Ex vivo safety literature on cold‐plasma on human corneal tissue [9, 16] or canine tissue [20] did not reveal any similar findings, nor did the case series of four volunteers [9] or in vivo studies in rabbits on corneal wound healing [21] and methicillin‐resistant *Staphylococcus aureus* keratitis [18].

In an in vivo study comparing two different builds of cold plasma jets for the treatment of fungal keratitis in a rabbit model, the authors found that a single strong jet caused more damage to the epithelium than a device with multiple microplasma jets [19]. Recently, Gilger et al. [22] conducted an in vivo safety study with cold plasma on rabbits, using different cold plasma builds without carrier gas (DBD: dielectric barrier discharge source). They observed focal corneal epithelial opacity on slit‐lamp examination and hyperechoic 2/3 of the superficial cornea on optical coherence tomography. These opacities had disappeared 24 h after DBD cold plasma treatment [22]. In our study, these white spots had also disappeared by the next follow‐up examination. We suspect that these are a type of “burn” caused by insufficient movement across the cornea—they could occur in areas with prolonged continuous ACP irradiation. This finding warrants further investigation with advanced ocular diagnostics, if they develop further in the treated animals.

The second advert reaction observed was that two dogs experienced pain directly after the ACP treatment. In one, topical anesthesia was insufficient, while the second was a hypersensitive dog (white French Bulldog with severe atopy, which also reacted to diamond burr debridement and several topical medications). This overall low number of painful animals shows that there should be no concerns using ACP therapy. However, topical anesthesia is essential for pain‐free ACP therapy. In the case series by Reitberger et al., none of the patient volunteers reported any pain during or after cold plasma treatment, although some found the jet stream of the ACP unpleasant [9]. When the effect of the topical anesthesia wore off, the patients felt a slight discomfort, which the authors believed was probably due to the disease itself [9].

After ACP treatment, several different adverse events were observed, the relationship of which to cold plasma is unclear. First, 2.5% of dogs (*n* = 7) treated for SCCED developed stromal infiltration during the course of ACP treatment, with onset occurring between 6 days after the first ACP treatment and 5 days after the sixth ACP treatment. Most of these dogs were brachycephalic breeds (four French Bulldogs, one Old English Bulldog, one Boxer, one Yorkshire Terrier), and all owners reported fairly sudden worsening of ocular symptoms. In one of the cases in which no bacterial culture was performed, cytology showed neutrophilic infiltration without bacteria. In other cases, repeated bacteriological culture was either negative (*n* = 2) or cultured bacteria were sensitive to initial antibiotics (*n* = 2). Two cases were not cultured a second time, with one sample showing deep infiltrate but healed epithelium. In addition, one French Bulldog treated with SCCED developed keratomalacia after the third ACP session. The initial SCCED preventive bacteriological sample in this case revealed *Enterobacter ludwigii* , a multi‐resistant pathogen that should actually be sensitive to ofloxacin, our first antibiotic in this case. The second bacteriological sample after the onset of keratomalacia revealed the same germ with the same sensitivity spectrum. However, the deterioration could not be stopped by additional antibiotics or anti‐collagenase treatments, including topical and systemic tetracycline therapy, topical autologous serum, topical acetylcysteine and corneal crosslinking, and the eye perforated and was enucleated at the owner's request. The development of stromal infiltrate, which may be sterile or infective, or keratomalacia is a known complication after SCCED treatment with various techniques [24] and is more common in brachycephalic breeds [24, 25, 26].

Secondly, in several animals (1.8%, three dogs and two rabbits) that had keratomalacia or infected stromal ulcers, the process could not be stopped by ACP treatment, and the animals went on to develop descemetocele and/or corneal perforations. We believe this adverse event is solely due to the fact that the treatment was started at a later stage of the disease (unintended development of a descemetocele) or that ACP was unable to stop the corneal process, and not the effect of the ACP itself. Although this was not part of this prospective safety study, the ACP treatment showed very promising results in the treatment of stromal infections and keratomalacia, so we assume that these were either animals that did not respond or that the ACP treatment was started too late.

The last short‐term side effect was observed in dogs treated with SCCED. Twelve of these dogs developed either epithelial tears after complete re‐epithelization (fluorescein‐negative cornea with missing epithelium‐stroma junction) or, in animals in which the SCCEDs had not yet completely healed centrally, a new tear developed at the periphery. It is questionable whether this was caused by superficial hyaline acellular zones of the stromal [27] remaining untouched during diamond burr debridement in these cases or whether the basement membrane showed delayed healing [28]. We suspect that these peripheral tears were caused by too rapid wound healing due to ACP treatment, as ACP promotes wound healing [2]. Another possibility is that these changes could occur in dogs treated only with conventional SCCED treatments, but follow‐up checks are not as frequent as in dogs treated with ACP (we usually perform standard rechecks after debridement for the first time after 10–14 days).

In the long‐term follow‐up part of this prospective study, only very few adverse events were observed. The most common, persistent corneal fibrosis and pigmentation, occurred almost exclusively in brachycephalic breeds (10/12). Four of these were French Bulldogs, three were Pugs, two were Shih‐Tzus, one was a Boxer and only two were dolichocephalic breeds: Podenco and Spitz. Increased scarring and pigmentation are common signs in dogs with brachycephalic ocular syndrome [29, 30, 31] and also occur when dogs are treated for aqueous‐deficient or evaporative dry eye disease [32]. Therefore, we believe that this is not an effect of ACP treatment, but an inherent characteristic of corneal healing in brachycephalic dogs. Three of the dogs developed glaucoma during long‐term follow‐up after ACP treatment. In one dog, it occurred after 163 days, and retinal detachment was diagnosed as well. In this case, despite further diagnostics, no etiological factor could be identified, and the dog was treated conservatively. The second dog was a perforation case in which secondary glaucoma and buphthalmos occurred 310 days after ACP therapy. The third case developed ocular hypertension 451 days after ACP therapy. This dog, a French Bulldog, was diagnosed with multiple iris melanomas, which were treated with transcorneal laser therapy prior to ACP application. This dog was treated medically and the eye was visual at the last follow‐up examination. Therefore, we believe that all of these glaucoma cases were merely coincidental occurrences following ACP therapy. All other long‐term adverse events occurred very rarely—only once in the dataset (retrobulbar process, episcleritis, SCCED and endothelial dystrophy) and are therefore very unlikely to be a consequence of ACP treatment.

The limitation of the present study is the small number of cases in cats and small mammals. This could lead to a bias in the feasibility of ACP treatment in clinical practice for those species. In cats, one in eight cases (12.5%) showed some distress during the procedure, which is high compared to eight cases in dogs (3.0%). By selecting more cooperative cats that may not have found the ACP jet stream unpleasant (none of the cats were irradiated under sedation like gabapentin or pregabalin), the feasibility of ACP treatment for cats with painful eye diseases may be lower in general.

In conclusion, ACP with the kINPen VET is clinically feasible and is safe to use in diseased corneas with few side effects and adverse events immediately after treatment and in the long‐term. It can be easily integrated into clinical treatment protocols as an adjunctive therapy for corneal diseases.

## Author Contributions

**Petr Soukup:** conceptualization, investigation, writing – original draft, methodology, visualization, validation, writing – review and editing, formal analysis, data curation. **Ingrid Allgoewer:** conceptualization, investigation, writing – original draft, methodology, validation, writing – review and editing, project administration, supervision, resources.

## Disclosure

The authors have not used AI to generate any part of the manuscript.

## Ethics Statement

The study was performed according to GERVO guidelines and was approved by the responsible authority (Landesamt für Gesundheit und Soziales, Berlin, Germany) under the registration number StN° 032–2023.

## Conflicts of Interest

The authors declare no conflicts of interest.

## Supporting information


**Table S1:** List of animals showing adverse reactions after ACP treatment.

**Table S2:** List of animals showing short‐term adverse events after ACP treatment.

**Table S3:** List of animals showing long‐term adverse events after ACP treatment.

## Data Availability

The data that supports the findings of this study are available in the Supporting information of this article.
