# Supplementary material for: Feasibility and Safety of Argon Cold Plasma Use as an Adjunctive Treatment for Corneal Disease in Dogs, Cats and Small Mammals: A Prospective Clinical Study
Source: Vet Ophthalmol. 2026 Jan 30;29(2):e70145. doi: 10.1111/vop.70145 (PMC12856724; doi:10.1111/vop.70145)
Supplement: Supplementary file 1 — Table S1: List of animals showing adverse reactions after ACP treatment. [file VOP-29-0-s002.docx]

| Breed | Age (years) | Follow-up (days) | Presenting complaint | Eye | Total Nr. of ACP treatments | Adverse reaction description |
| --- | --- | --- | --- | --- | --- | --- |
| French Bulldog | 7.8 | 26 | SCCED, EDED OU | OU | 3 | Painful for several hours after ACP treatment with blepharospasm and ocular discharge. This effect resolved approximately 18 hours after ACP. This episode repeated after each ACP treatment (three in total, where OD healed and OS was later lost to follow-up). White coat dog with severe atopy. |
| Mixed-breed | 12.9 | 337 | SCCED OD | OD | 3 | Painful for several hours after the second ACP treatment, blepharospasm. It was found out that the dog did not receive sufficient topical anesthesia (only one drop of topical anesthesia was applied approximately 5 minutes before ACP treatment. First and third ACP treatments were unremarkable. |
| French Bulldog | 8.6 | 15 | SCCED OS, EDED OU | OS | 3 | Whitish spots in the corneal epithelium and anterior stroma that did not retain fluorescein stain developed directly after the 3rd ACP treatment. They were resolved by the next recheck six days later. |
| French Bulldog | 4.9 | 23 | SCCED OS | OD | 3 | Whitish spots in the corneal epithelium and anterior stroma that did not retain fluorescein stain developed directly after the 3rd ACP treatment. They were resolved by the next recheck fourteen days later. |
| French Bulldog | 6.7 | 28 | infected stromal ulcer OD | OD | 2 | Whitish spots in corneal epithelium and anterior stroma with faint fluorescein uptake centrally within the spots developed directly after the 2nd and 3rd ACP treatment. They were resolved by the next recheck seven days later. |
| French Bulldog | 4.1 | 175 | infected stromal ulcer OS | OS | 3 | Whitish epithelial eschars developed at the edges of the ulcer after 1st ACP treatment. These could be partially removed with a cotton tip applicator afterwards. Whitish spots were resolved by the next recheck two days later, even though ulcer was not yet healed. |

Supplementary Table 1: List of animals showing adverse reactions after ACP treatment

Abbreviations: spontaneous chronic corneal epithelial defect (SCCED), evaporative dry eye disease (EDED), argon cold plasma (ACP)
