# Supplementary material for: Feasibility and Safety of Argon Cold Plasma Use as an Adjunctive Treatment for Corneal Disease in Dogs, Cats and Small Mammals: A Prospective Clinical Study
Source: Vet Ophthalmol. 2026 Jan 30;29(2):e70145. doi: 10.1111/vop.70145 (PMC12856724; doi:10.1111/vop.70145)
Supplement: Supplementary file 2 — Table S2: List of animals showing short‐term adverse events after ACP treatment. [file VOP-29-0-s001.docx]

Supplementary table 2: List of animals showing short-term adverse events after ACP treatment

| Breed | Age (years) | Follow-up (days) | Presenting complaint | Eye | Total Nr. of ACP treatments | Adverse event description: Short term |
| --- | --- | --- | --- | --- | --- | --- |
| French Bulldog | 5.7 | 274 | SCCED OD | OD | 9 | Initial corneal culture yielded *S. pseudintermedius* (sensitive to antibiotic treatment that was started after first ACP treatment). Stromal infiltrate has developed 5 days after 6th ACP treatment. No cytology or additional bacterial culture was performed. Change of antibiotic treatment. Healed uneventfully after two more ACP treatments. |
| French Bulldog | 7.8 | 372 | SCCED OD | OD | 3 | Initial corneal culture was negative. Stromal infiltrate developed 6 days after 3rd ACP treatment. Cytology showed neutrophilic infiltrate and no bacteria were observed. Antibiotic treatment was not changed; autologous serum was added to the therapy. No more ACP treatments were performed. Healed uneventfully. |
| French Bulldog | 10.0 | 86 | SCCED OS, ADDE OU | OS | 6 | Initial corneal culture was negative. Stromal infiltrate developed 6 days after 1st ACP treatment. Cytology was not performed, new corneal culture yielded *Streptococcus hominis*, sensitive to initial antibiotic. Antibiotic treatment was changed before culture resulted were received (also sensitive). Healed uneventfully after another five ACP treatments. |
| French Bulldog | 7.6 | 21 | SCCED OD | OD | 5 | Initial corneal culture was negative. Stromal infiltrate developed 5 days after 2nd ACP treatment. Cytology was not performed; new corneal culture was negative as well. Antibiotic treatment was changed before culture resulted were received. Healed uneventfully after another 3 ACP treatments. |
| Boxer | 9.2 | 410 | SCCED OD | OD | 6 | Initial corneal culture was negative. Stromal infiltrate developed 7 days after 2nd ACP treatment. Cytology was not performed; new corneal culture was negative as well. Antibiotic treatment was changed before culture resulted were received. Healed uneventfully after another 4 ACP treatments. |
| Old English Bulldog | 7.9 | 133 | SCCED OS | OS | 4 | Initial corneal culture was negative. Stromal infiltrate developed 3 days after 3rd ACP treatment. Cytology was not performed, new corneal culture yielded *S. pseudintermedius and S. capitis*, both sensitive to initial antibiotic treatment. Antibiotic treatment was changed before culture resulted were received. Healed uneventfully after another 1 ACP treatment. |
| Yorkshire Terrier | 11.4 | 27 | SCCED OS, EDED OU | OS | 1 | No bacterial culture was performed due to pretreatment with ofloxacin. Stromal infiltrate developed 10 days after 1st ACP therapy, nevertheless, the epithelium was healed and infiltrate disappeared without any intervention two weeks later. |
| French Bulldog | 7.7 | 24 | SCCED OD | OD | 4 | Initial corneal culture yielded high amount of *Enterobacter ludwigii* which was supposed to be sensitive to initial antibiotic treatment. Keratomalacia started developing 3 days after 3rd ACP treatment. Cytology was not performed, new corneal culture yielded again high amount of *Enterobacter ludwigii*, which was again sensitive to initial antibiotics as well as the antibiotic ointment that was added into therapy regimen. The melting process did not stop even with anti-collagenase medication, corneal crosslinking and UV-C treatment and perforated 24 days after initial presentation. Owner decided for enucleation procedure. |
| Parson Russel Terrier | 12.1 | 63 | infected deep stromal ulcer OD, ADDE OU | OS | 2 | Initial corneal culture yielded high number of beta-hemolytic *Streptococci* which was supported by cytology. *us* which was sensitive to initial antibiotic treatment. Stromal ulcer could not be halted with medical therapy and two ACP treatments and the stroma melted until descemetocele, which in the end did not perforate and was managed medically until healed. Uncontrolled Diabetes mellitus. |
| French Bulldog | 10.0 | 1 | keratomalacia OS, ADDE OU | OS | 1 | Large-diameter keratomalacia that perforated one day after initial presentation. Owner decided for enucleation procedure. |
| French Bulldog | 5.9 | 62 | keratomalacia OD | OD | 3 | Large-diameter keratomalacia which developed into large-diameter descemetocele and perforated one day after 3rd ACP treatment. Initially, owner was reluctant to perform surgery. |
| Lionhead rabbit | 7.2 | 104 | keratomalacia OS | OS | 6 | Large-diameter keratomalacia that perforated eleven days after 6th ACP treatment. Healed after Descemet’s membrane resection under topical anesthesia. |
| Holland lop rabbit | 10.1 | 112 | infected stromal ulcer OS | OS | 3 | Large-diameter keratomalacia that developed into large-diameter descemetocele and perforated thirteen days after 3rd ACP treatment. Initial corneal culture yielded high amount of multi-resistant *Pseudomonas aeruginosa*. Healed conservatively after additional ACP treatment. |
| French Bulldog | 7.5 | 549 | SCCED OD, ADDE OU | OD | 6 | Peripheral epithelial tear in centrally not-yet healed SCCED after 4th ACP treatment, healed uneventfully after six ACP treatments. |
| French Bulldog | 9.6 | 522 | SCCED OS, EDED OU | OS | 4 | Peripheral epithelial tear in centrally not-yet healed SCCED after 3rd ACP treatment, healed uneventfully after four ACP treatments. |
| French Bulldog | 9.1 | 30 | SCCED OS, EDED OU | OS | 5 | Peripheral epithelial tear in centrally not-yet healed SCCED after 2nd ACP treatment, healed uneventfully after five ACP treatments. |
| French Bulldog | 7.2 | 68 | SCCED OD | OD | 6 | Peripheral epithelial tear in centrally not-yet healed SCCED after 2nd ACP treatment, healed uneventfully after five ACP treatments. |
| French Bulldog | 8.5 | 212 | SCCED OD, EDED OU | OD | 6 | Developed epithelial tear after complete reepithelization after 4 ACP treatments, healed after another two ACP treatments. |
| French Bulldog | 7.1 | 469 | SCCED OD, EDED OU | OD | 7 | Developed epithelial tear after complete reepithelization after 3 ACP treatments, healed after another four ACP treatments. |
| French Bulldog | 6.2 | 415 | SCCED OS | OD | 7 | Developed epithelial tear after complete reepithelization after 3 ACP treatments, healed after another four ACP treatments. |
| French Bulldog | 6.6 | 119 | SCCED OD, ADDE OU | OD | 4 | Developed epithelial tear after complete reepithelization after 2 ACP treatments, healed after another two ACP treatments, two new debridements and third eyelid flap. |
| French Bulldog | 8.1 | 103 | SCCED OS, EDED OU | OS | 5 | Developed peripheral epithelial tear after complete reepithelization after 4 ACP treatments and several days later developed central epithelial tear, healed after another one ACP treatments and a new debridement. |
| Welsh Corgi Pembroke | 8.5 | 147 | SCCED and CED OS | OS | 4 | Developed epithelial tear after complete reepithelization after 4 ACP treatments, healed later uneventfully. |
| Boxer | 9.1 | 120 | SCCED OS | OS | 5 | Developed epithelial tear after complete reepithelization after 4 ACP treatments, healed after one another ACP treatment uneventfully. |
| Boxer | 7.9 | 53 | SCCED OS | OS | 6 | Peripheral epithelial tear in centrally not-yet healed SCCED after 2nd ACP treatment, healed uneventfully after additional four ACP treatments and new debridement. |

Abbreviations: spontaneous chronic corneal epithelial defect (SCCED), evaporative dry eye disease (EDED), aqueous dry eye disease (ADDE), argon cold plasma (ACP)
