# Supplementary material for: Feasibility and Safety of Argon Cold Plasma Use as an Adjunctive Treatment for Corneal Disease in Dogs, Cats and Small Mammals: A Prospective Clinical Study
Source: Vet Ophthalmol. 2026 Jan 30;29(2):e70145. doi: 10.1111/vop.70145 (PMC12856724; doi:10.1111/vop.70145)
Supplement: Supplementary file 3 — Table S3: List of animals showing long‐term adverse events after ACP treatment. [file VOP-29-0-s003.docx]

Supplementary table 3: List of animals showing long-term adverse events after ACP treatment

| Breed | Age (years) | Follow-up (days) | Presenting complaint | Eye | Total Nr. of ACP treatments | Adverse event description: Long term |
| --- | --- | --- | --- | --- | --- | --- |
| French Bulldog | 6.7 | 164 | SCCED OD | OD | 5 | SCCED healed after 23 days, pigmentary keratitis was developed by day 31, improved only partially with topical tacrolimus and dexamethasone by the last recheck 15 months later |
| French Bulldog | 10.1 | 273 | SCCED OU, ADDE OU | OS | 9 | Developed chronic pigmentary keratitis during the SCCED healing time which required nine ACP treatments, additional debridement and temporary tarsorrhaphy in both eyes. Improved only partially with topical tacrolimus 6 months later. ADDE patient. |
| French Bulldog | 10.1 | 273 | SCCED OU, ADDE OU | OD | 9 | Same dog as above, second eye. |
| French Bulldog | 9.2 | 82 | SCCED OD | OD | 4 | SCCED healed after 42 days, pigmentary keratitis was developed by that time, improved only partially with topical tacrolimus by the last recheck 1.5 month later, lost to follow-up. |
| Boxer | 8.6 | 619 | SCCED OD, EDED OU | OS | 6 | SCCED healed after 26 days, pigmentary keratitis was developed by day 85, improved only partially with topical tacrolimus and dexamethasone by the last recheck 12 months later. |
| Podenco | 14.6 | 298 | SCCED OS | OS | 4 | Developed chronic fibrosis and pigmentary keratitis during the SCCED healing time which required four ACP treatments, additional debridement, third eyelid flap and contact lens. Improved minimally with topical tacrolimus and dexamethasone therapy 10 months later. |
| Spitz | 18.7 | 448 | infected stromal ulcer OD | OD | 3 | Stromal ulcer healed after three ACP treatments in 8 days. Developed chronic fibrosis and pigmentary keratitis in that eye 6 months later. Improved minimally with topical hyaluronic acid therapy 1.5 year later. Senile canine endothelial dystrophy patient. |
| Shih-Tzu | 5.6 | 441 | infected stromal ulcer OS, ADDE OU | OS | 3 | infected ulcer healed after 20 days, pigmentary keratitis was developed by day 30, worsened under topical tacrolimus and dexamethasone in next 14 month. Cornea completely pigmented by the last recheck. ADDE patient. |
| Pug | 11.8 | 135 | infected stromal ulcer OS, ADDE OU | OS | 3 | Stromal ulcer healed after three ACP treatments in 16 days. Developed chronic fibrosis and pigmentary keratitis by day 44. Improved only partially with topical ciclosporin therapy 3 months later. ADDE patient. Lost to follow-up. |
| Shih-Tzu | 13.1 | 72 | infected stromal ulcer OS, ADDE OU | OS | 8 | Developed chronic fibrosis and pigmentary keratitis during large infected stromal ulcer healing time which required eight ACP treatments. ADDE patient. Improved minimally with 5-week topical ciclosporin, lost to follow-up. |
| Pug | 12.0 | 176 | infected stromal ulcer OS, ADDE OU | OS | 5 | Developed chronic fibrosis and pigmentary keratitis during infected stromal ulcer healing time which required five ACP treatments. Worsened under topical tacrolimus over next 5 months OU. Cornea completely pigmented by the last recheck. ADDE patient. |
| Pug | 12.0 | 342 | corneal perforation with iris prolapse OD, ADDE OU | OD | 4 | Marked pigmentary keratitis prior to corneal perforation. Perforation healed conservatively after 4 ACP treatments. Pigmentation worsened under topical dexamethasone, ciclosporin and tacrolimus over next 10 months OU. Cornea completely pigmented by the last recheck. ADDE patient. |
| French Bulldog | 7.4 | 560 | SCCED OS, ADDE OU | OS | 4 | Developed glaucoma 451 days after ACP therapy. Diagnosed with multiple iris melanomas and treated by transcorneal laser over large portion of the iris prior to ACP therapy. Further medical management, visual at the last recheck. |
| Australian shepherd | 7.5 | 262 | SCCED OS | OS | 4 | Developed glaucoma and retinal detachment 163 days after first ACP therapy. No trauma reported. Despite further internal medicine workup, no etiologic cause was found out and the dog was treated conservatively for two months, then lost to follow-up. |
| English Bulldog | 3.8 | 315 | corneal perforation OS | OS | 1 | Developed secondary glaucoma and buphthalmos 310 days after ACP therapy for perforation. |
| Mixed-breed | 10.7 | 183 | keratomalacia OD | OD | 3 | Under long-term treatment for left-sided masticatory muscle myositis, was diagnosed with trigeminal nerve paralysis after ACP treatment and healing of the melting ulcer, which was most likely present before the keratomalacia |
| Parson Russel Terrier | 11.1 | 241 | infected stromal ulcer OS | OS | 3 | Developed SCCED 298 days after ACP therapy. |
| Mixed-breed | 12.9 | 337 | SCCED OD | OD | 3 | Developed diffuse episcleritis 108 days after the first ACP therapy. Under topical tacrolimus therapy under control at the last recheck 11 months post ACP treatment. |
| Boxer | 9.2 | 410 | SCCED OD, EDED OU | OD | 6 | Developed canine endothelial dystrophy 410 days after ACP treatment (Boxer). |
| French Bulldog | 9.0 | 400 | SCCED OS | OS | 4 | Developed retrobulbar process 400 days after ACP treatment, referral to imaging and following surgical therapy. Lost to follow-up. |

Abbreviations: spontaneous chronic corneal epithelial defect (SCCED), evaporative dry eye disease (EDED), aqueous dry eye disease (ADDE), argon cold plasma (ACP)
